# Enhanced visualization of flat gastric dysplasia by chromoendoscopy using an acetic acid-indigocarmine mixture

**DOI:** 10.1055/a-2832-6161

**Published:** 2026-03-26

**Authors:** Masaya Iwamuro, Katsunori Matsueda, Motoyuki Otsuka

**Affiliations:** 1 12997 Department of Gastroenterology and Hepatology, Graduate School of Medicine, Dentistry, and Pharmaceutical Sciences, Okayama University, Okayama, Japan

A 76-year-old man with a history of diabetes mellitus, hypertension, hyperlipidemia, and prostate cancer underwent annual surveillance esophagogastroduodenoscopy after successful *Helicobacter pylori* eradication. During routine endoscopy, a subtle lesion was suspected at the lesser curvature of the gastric angle, and the patient was referred for further evaluation.

**Fig. 1:**
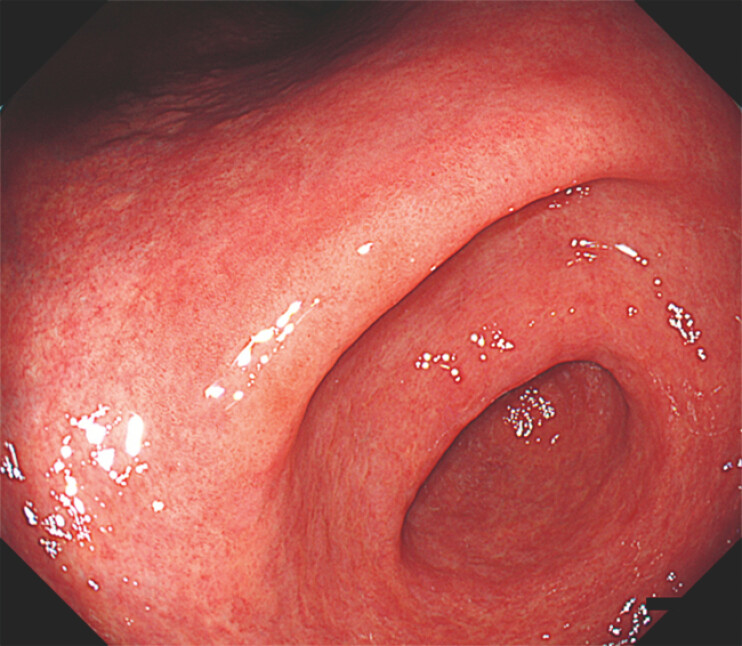
White-light esophagogastroduodenoscopy showing a flat lesion at the lesser curvature at the gastric angle. The lesion demonstrates only a faint whitish discolouration without surface irregularity, making recognition challenging under white-light imaging.

**Fig. 2:**
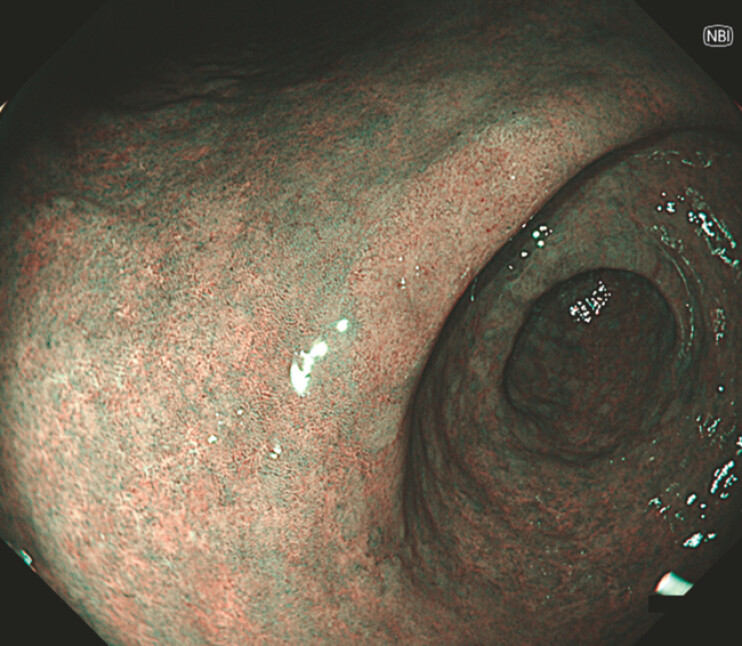
Narrow-band imaging reveals a brownish area corresponding to the flat dysplastic lesion, allowing visualization.

**Fig. 3:**
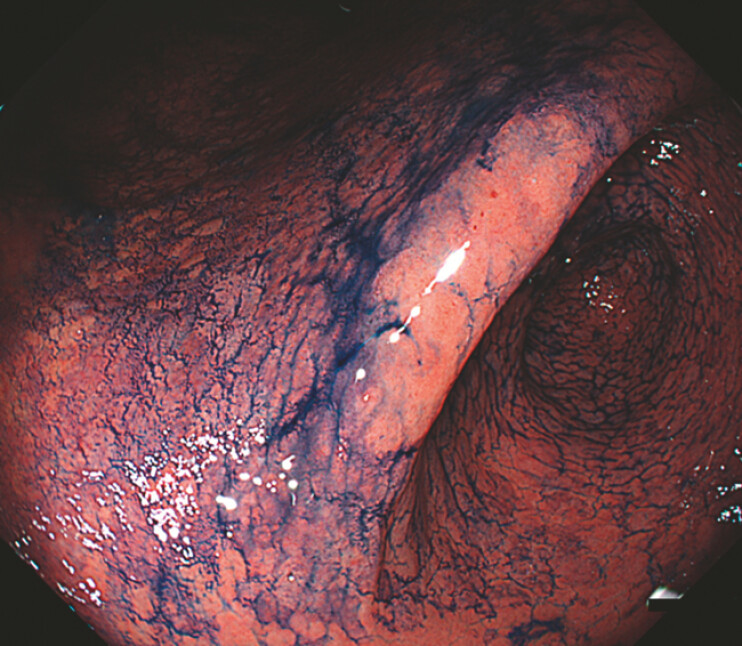
Chromoendoscopy using an acetic acid–indigocarmine mixture. The surrounding non-neoplastic mucosa shows acetowhitening with diffuse blue indigocarmine dye pooling on the mucosal surface, whereas the dysplastic epithelium repels the dye and appears as a distinct pink area with sharp margin delineation.

Esophagogastroduodenoscopy revealed a flat, approximately 20-mm dysplastic lesion. Under white light imaging, the lesion showed only a faint whitish discolouration without surface irregularities, making recognition challenging ( Video 1 ; Fig. 1 ). Narrowband imaging revealed a brownish area ( Fig. 2 ), and magnifying observations demonstrated a clear demarcation line with irregular microvascular and microsurface patterns consistent with gastric dysplasia. To improve lesion detection and margin delineation, an acetic acid–indigocarmine mixture (AIM) was sprayed ( Fig. 3 ). The mixture consisted of 15 mL of distilled water containing dimethicone, 15 mL of 1.5% acetic acid solution, and 10 mL of indigocarmine. The surrounding mucosa exhibited acetowhitening with diffuse blue indigocarmine dye pooling on the mucosal surface, whereas the dysplastic epithelium repelled the dye and appeared as a distinct pink area, sharply contrasting with the background mucosa.

White-light imaging demonstrated a subtle flat lesion with a minimal colour change at the lesser curvature of the gastric angle, followed by narrow-band imaging and acetic acid–indigocarmine mixture chromoendoscopy, which clearly delineated the lesion margins.Video 1

**Fig. 4:**
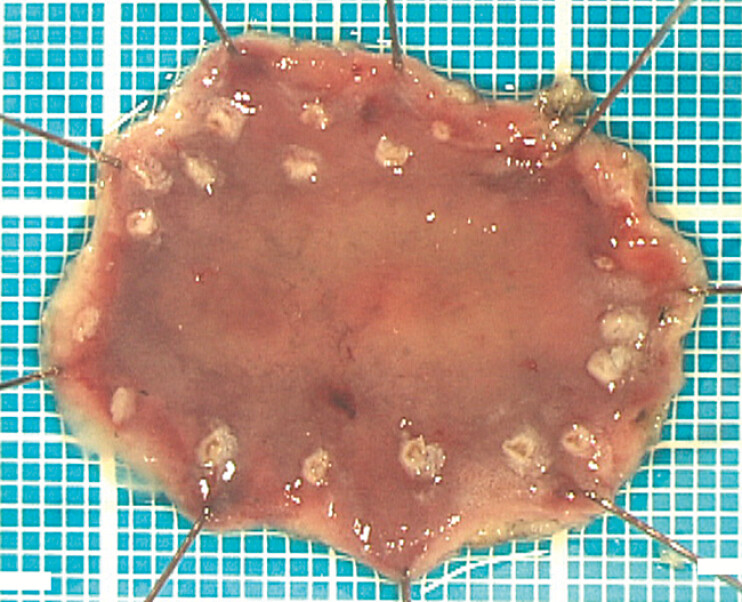
Resected specimen after endoscopic submucosal dissection, demonstrating complete en bloc resection with negative horizontal and vertical margins (HM0 and VM0). Upon gross inspection, the lesion appears as a slightly whitish area.

Endoscopic submucosal dissection was performed, achieving *en bloc* resection with negative horizontal and vertical margins (HM0 and VM0), confirming curative treatment ( Fig. 4 ).

The acetic acid–indigocarmine mixture enhances mucosal contrast by combining acetowhitening and differential surface dye retention 1 2 3 . Normal gastric mucosa allows the indigocarmine dye to pool on the mucosal surface and appears diffusely blue, whereas neoplastic epithelium tends to repel the dye and shows reduced acetowhitening, resulting in a pink appearance. This optical contrast is particularly advantageous for detecting flat gastric lesions with minimal baseline colour changes. This case highlights the utility of AIM-assisted chromoendoscopy for the accurate identification and margin delineation of flat gastric dysplasia.

Endoscopy_UCTN_Code_CCL_1AB_2AD_3AB Endoscopy_UCTN_Code_TTT_1AO_2AB
